# Is non-invasive ventilation effective in improving the exercise capacity in patients with cardiac heart failure?: A randomised crossover trial

**DOI:** 10.1371/journal.pone.0327399

**Published:** 2025-07-07

**Authors:** Guillermo Mazzucco, Rodrigo Torres-Castro, Leonardo Intelangelo, Ana Lista-Paz, Juan Pablo Escalante, Lore Zumeta-Olaskoaga, Gonzalo Veiga, Ane Arbillaga Etxarri

**Affiliations:** 1 Deusto Physical TherapIker, Department of Physiotherapy, Faculty of Health Sciences, University of Deusto, Donostia-San Sebastián, Spain; 2 Cardiopulmonary and Metabolic Rehabilitation Unit, Ammma, Donostia San-Sebastián, Spain; 3 Health and Rehabilitation Department, University of Gran Rosario, Rosario, Argentina; 4 Instituto Cardiovascular de Rosario, Rosario, Argentina; 5 Department of Physical Therapy, University of Chile, Santiago, Chile; 6 University Center for Assistance, Teaching and Research (CUADI), University of Gran Rosario, Rosario, Argentina; 7 The University of A Coruña, Faculty of Physiotherapy, A Coruña, España; 8 Biogipuzkoa Health Research Institute, Donostia-San Sebastián, Spain; DM Wayanad Institute of Medical Sciences, INDIA

## Abstract

**Introduction:**

Heart failure (HF) is a prevalent global health issue, characterized by the heart’s inability to effectively pump or fill with blood, leading to inadequate cardiac output. Despite advances in medical treatments, exercise intolerance remains a significant challenge, impacting their quality of life and contributing to frequent hospitalizations. Recent studies suggest that non-invasive ventilation (NIV) may further enhance exercise performance by reducing ventilatory workload and fatigue. However, limited research has directly compared different ventilatory modes during exercise in patients with heart failure. This study aims to evaluate the effects of two NIV devices on exercise capacity.

**Methods:**

A randomised crossover trial was conducted in patients with HF, reduced ejection fraction (≤ 40%), New York Heart Association functional class I-III and clinically stable. All participants underwent an initial assessment followed by an incremental exercise test to determine maximum aerobic velocity. They were then randomized to perform three constant work rate tests on separate days under three conditions: (1) with Continuous Positive Airway Pressure (CPAP), (2) with pressure support (PS) and (3) without NIV. The primary outcome was time to exhaustion. Key physiological variables were recorded during each test. Participants were recruited and completed all testing between April 29 and July 22, 2022. ClinicalTrials.gov registration number: NCT05433610.

**Results:**

A total of 11 patients (mean age: 67 ± 9.6 years) completed the study. Exercise duration was significantly longer in the pressure support group (9.8 ± 6.2 minutes) compared to the CPAP group (8.9 ± 6.0 minutes) and the control group (7.3 ± 6.2 minutes) (p = 0.043). No significant differences were found in average heart rate (HR), final HR, or oxygen saturation (SpO₂) between the groups (p > 0.05). Similarly, dyspnea and leg fatigue (modified Borg scale) showed no statistically significant differences between conditions (p > 0.05).

**Conclusion:**

The use of NIV, particularly the PS mode, during exercise significantly improved exercise duration in patients with HF compared to CPAP or no ventilatory support.

**Clinical Trial Registration:**

ClinicalTrials.gov Identifier: NCT05433610

## Introduction

Heart failure (HF) is a clinical syndrome characterized by the heart’s inability to pump or fill with blood effectively, leading to inadequate cardiac output and compensatory neurohormonal activation and increased left ventricular filling pressure [1]. This condition represents a major global health burden, with an estimated 64.3 million individuals affected worldwide in 2017 [2]. In the United States alone, approximately 6.7 million people over the age of 20 have HF, a number projected to rise to 11.4 million by 2050 [3].

The management of HF includes pharmacological treatments (ACE inhibitors, beta-blockers, diuretics) [4], lifestyle modifications (low-sodium diet, regular exercise, weight management) [5], and medical interventions such as implantable cardioverter defibrillators (ICDs), cardiac resynchronization therapy (CRT), left ventricular assist devices (LVADs), and heart transplantation [6]. Additionally, exercise training is strongly recommended as a key non-pharmacological therapy [7].

Clinical guidelines endorse exercise rehabilitation for clinically stable HF patients, regardless of their ejection fraction, emphasizing its safety and effectiveness in improving quality of life, functional capacity, and reducing HF-related hospitalizations [8,9]. An Expert Panel emphasize that exercise training and other components of cardiac rehabilitation (CR) are both safe and beneficial for patients with HF and these interventions lead to significant improvements in quality of life, functional capacity, exercise performance, and a reduction in HF-related hospitalizations [10].

Non-invasive ventilation (NIV) has emerged as a promising adjunct to exercise training in patients with HF. By improving ventilatory mechanics, reducing respiratory discomfort, and decreasing cardiac workload, NIV may enhance oxygen delivery and exercise tolerance [11–13]. Although several studies have explored its benefits, direct comparisons between different NIV modalities during exercise remain limited [14,15]. Further research could help refine personalized rehabilitation strategies, allowing patients to achieve higher exercise intensities and improved functional exercise capacity.

Therefore, the aim of this study was to evaluate and compare the effects of Continuous Positive Airway Pressure (CPAP) and Pressure Support (PS) on exercise performance in patients with HF with reduced ejection fraction (HFrEF). Unlike previous studies, we used a randomized crossover design to directly assess both NIV modalities under controlled conditions during exercise. This approach provides novel insights that may inform more personalized and effective rehabilitation strategies for this population.

## Materials and methods

### Subjects and design

Study preregistration, including original hypothesis, description of primary and secondary outcomes, and sample size consideration, is available at ClinicalTrials.gov (identifier: NCT05433610). For this crossover randomised controlled trial patients diagnosed with HFrEF were recruited between April 29, 2022, and July 22, 2022. All participants completed their testing procedures within this same period, typically over four separate visits. Eligible participants were required to meet the following inclusion criteria: (1) age ≥ 18 years, (2) ejection fraction ≤ 40% as demonstrated by echocardiography (within the last six months), (3) New York Heart Association (NYHA) functional class I – III and (4) clinical stability, defined as no hospitalizations in the four weeks prior to study initiation, Patients were excluded if they had at least one of the following conditions: (1) respiratory disease (Chronic obstructive pulmonary disease, interstitial lung disease or pulmonary hypertension) (2) unstable angina or significant arrhythmias (hemodynamic instability or recent medical intervention, such as sustained ventricular tachycardia, high-degree AV block, or uncontrolled atrial fibrillation (>100 bpm at rest)) (3) myocardial infarction within the last three months, (4) primary valve disease, or anemia (hemoglobin < 13 g/dl for men or < 12 g/dl for women), (5) current smokers or ex-smokers of less than one year, (6) cognitive impairment preventing accurate understanding of evaluations and/or (7) any neuromuscular or osteoarticular condition limiting test performance.

During the initial assessment, all patients were confirmed to be clinically stable and appropriately managed with medical therapy at least two months. An independent physician conducted the evaluations. All volunteers received both written and verbal descriptions of the procedures and provided signed written informed consent before participation. No exemptions from informed consent were granted by the ethics committee. The study was approved by the Bioethics Committee of Hospital Italiano (approval no. 30/21), and the Declaration of Helsinki was respected.

This randomized crossover trial is reported following the Consolidated Standards of Reporting Trials (CONSORT) guidelines for randomized trials, including its extension for crossover trials, to ensure methodological transparency and reproducibility [16].

### Experimental procedure

#### Exercise testing and non-invasive ventilation protocol.

During subsequent visits, participants underwent a ramp-incremental exercise test followed by three high-intensity constant work rate tests (CWRT) at 85% of the maximal aerobic speed reached during the incremental test. The order of the three CWRT conditions was randomized using a table of random numbers and included: 1) CWRT with Continuous Positive Airway Pressure (CPAP), 2) CWRT with Pressure Support (PS), and 3) CWRT without non-invasive ventilation (Control group). To minimize selection bias, the allocation sequence was concealed from the investigators enrolling participants until the moment of assignment. Randomization was managed by an independent third-party researcher, and participants were blinded to the NIV mode applied, while assessors were aware of the intervention during testing.

#### Incremental exercise test protocol.

Participants completed a ramp-incremental treadmill test to determine their maximal aerobic speed and maximum slope achieved. All evaluations were conducted by cardiologists from the cardiovascular rehabilitation unit, following American Heart Association guidelines [17].

After a standardized warm-up phase, the incremental protocol began at 3 km/h, with speed increasing by 0.5 km/h every minute until volitional exhaustion. The test was terminated if participants exhibited any of the following clinical signs or symptoms: angina pectoris (subjective chest pain consistent with myocardial ischemia), severe dyspnea (Borg scale ≥ 9), nausea or presyncope, cyanosis (noted by clinician), a drop in oxygen saturation below 90%, or arrhythmias identified by real-time ECG monitoring.

Maximal aerobic speed was defined as the highest speed and slope tolerated for a full 1-minute stage. Data were collected every minute during exercise and at 1, 2, 3, and 6 minutes post-exercise recovery. Monitored variables included blood pressure (BP), oxygen saturation (SpO₂), heart rate (HR), dyspnea, and lower limb fatigue, assessed using the modified Borg scale.

#### CWRT protocol and NIV administration.

Following maximal exercise testing, participants were randomly assigned to perform three CWRT sessions on separate days, with a minimum 48-hour rest period between sessions. Exercise intensity was set at 85% of the maximal aerobic speed and slope achieved during the incremental test. The primary outcome was time to exhaustion (Tlim).

Each CWRT session was performed under one of three conditions: (1) CPAP condition, where Continuous Positive Airway Pressure (CPAP) was set at 5 cmH₂O (ResMed AirCurve 10); (2) PS condition, where Pressure Support (PS) was applied with an inspiratory positive airway pressure (IPAP) of 10 cmH₂O and an expiratory positive airway pressure (EPAP) of 5 cmH₂O (ResMed AirCurve 10) and (3) Control group, in which participants exercised without NIV. In all sessions, NIV was administered via an oronasal mask.

Before each CWRT, participants rested for 5 minutes to establish baseline values, including HR, BP, SpO₂, dyspnea, and lower limb fatigue (Borg scale, 0–10). During this period, NIV was applied in the CPAP and PS conditions, while in the control condition, participants rested without NIV.

**Fig 1:**
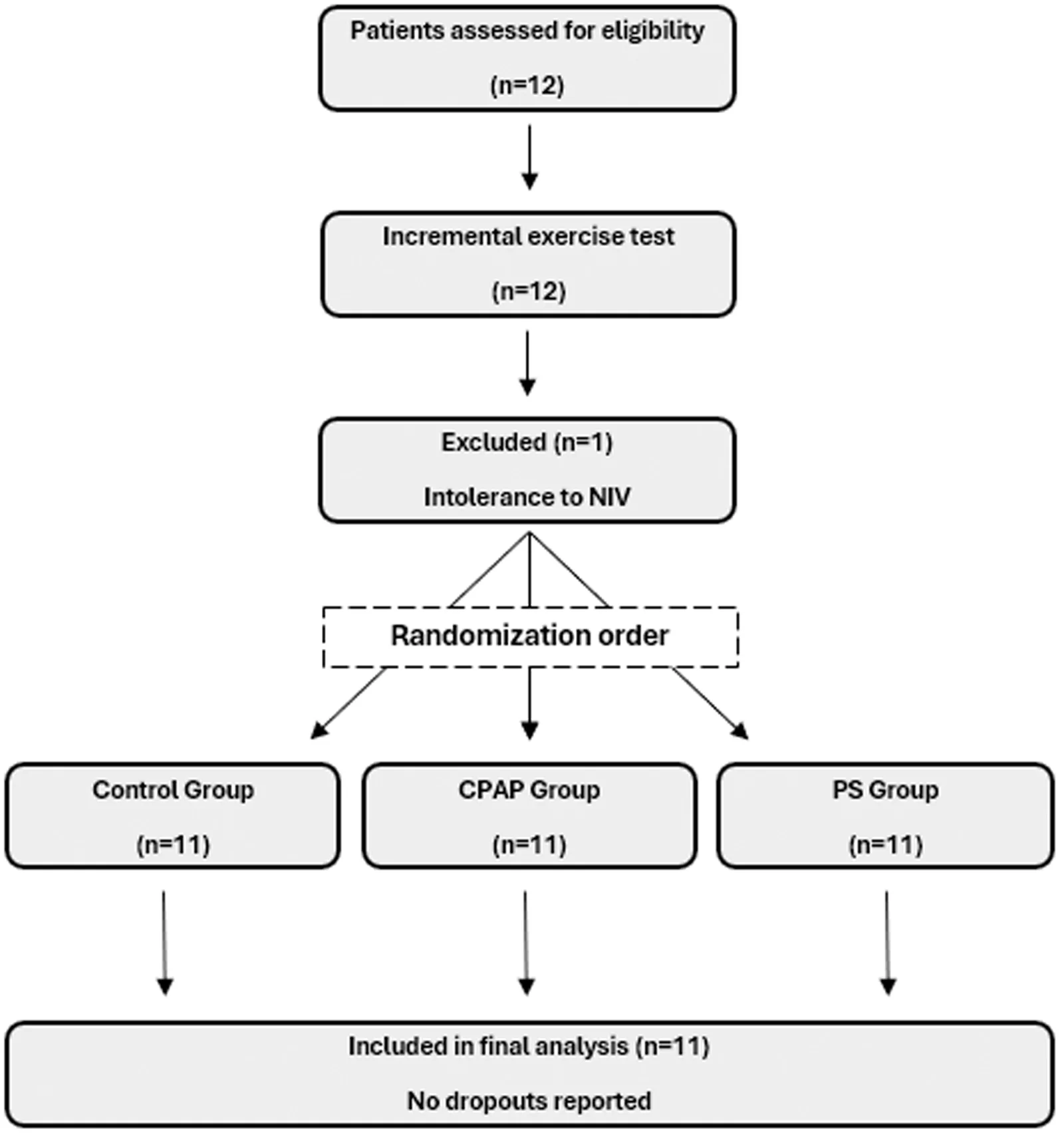
Flowchart of participants through the study, including enrolment, randomization, allocation, and analysis. Abbreviations: NIV, non-invasive ventilation; CPAP, continuous positive airway pressure; PS, pressure support.

During the CWRT, HR, SpO₂, dyspnea, and lower limb fatigue were recorded every minute. These variables were also assessed for 3 minutes post-exercise. Additionally, participants reported their overall comfort with NIV and provided reasons for stopping the exercise (e.g., dyspnea or fatigue). Further details on randomization are presented in Fig 1.

### Statistical analysis

The sample size calculation was based on the maximum time achieved in the CWRT, selected as selected as the primary outcome due to its high sensitivity in detecting changes following both pharmacological and non-pharmacological interventions, as well as its widespread use in clinical trials. Based on previous literature, a minimum detectable difference of 100 seconds was established [15]. The sample size was determined using a paired t-test for repeated measures, considering a difference of 1.4 minutes (84 seconds) among conditions (PS, CPAP, and no ventilatory support). A power analysis performed using G-Power (3.1.9.2 software) indicated that a sample of 12 participants would be required to achieve a statistical power of 0.80, with an alpha level of 0.05.

A descriptive analysis was conducted to explore and summarize the main characteristics of the data. Quantitative variables were reported with means and standard deviations or, in the case of asymmetric distributions, with median and interquartile range (IQR). Qualitative or categorical variables were presented as absolute and relative frequency distributions (percentages). To study differences in the outcome variable across different study groups, repeated measures ANOVA or the Friedman test was used, depending on whether the data met the normality assumption. For pairwise comparisons, the Wilcoxon test was applied. All statistical analyses were performed using the open-source statistical software R [18] version 4.4.1 with a significance level of 95%. Specifically, the tidyverse [19] and ggplot2 [20] packages were used for data handling and data visualization, the compareGroups [21] package was used for the initial descriptive statistical analysis and rstatix [22] package for hypothesis testing.

## Results

A total of 11 patients were included in the study after excluding one patient who was unable to adapt to NIV and experienced intolerance during the aerobic evaluation. The baseline characteristics of the sample are summarized in Table 1.

**Table 1:** Clinical characteristics of the study patients, mean (sd) are shown for continuous variables and frequencies (%) for categorical variables (n=11).

Anthropometric characteristics mean (SD)	
Males/females	8 (73%)/ 3 (27%)
Age (years)	67 ± 9.6
Height (cm)	173 ± 7.9
Body mass (kg)	81.5 ± 11.5
Body mass index (kg/m²)	27.2 ± 2.5
HR (bpm)	65.7 ± 9.9
SpO_2_ (%)	98 ± 0.9
SBP (mmHg)	118.18 ± 13.47
DBP (mmHg)	63.64 ± 8.09
**Echocardiography** mean (SD) [min, max]	
LVEF (%)	30.3 ± 7.3
**Etiology of heart failure**	
Ischemic	9 (81.8%)
Non-ischemic	2 (18.2%)
**Functional class (NYHA)**	
I	2 (18%)
II	8 (73%)
III	1 (9%)
IV	0 (0%)
**Medications**	
Diuretic	9 (81.8%)
Digitalis	5 (45.5%)
Carvedilol	10 (90.9%)
Angiotensin-converting enzyme inhibitor	6 (54.5%)
**Maximal exercise incremental test** mean (SD) [min, max]	
Velocity (Km/h)	7 ± 1.6
HR (bpm)	122 ± 25.4
SpO_2_ (%)	96.5 ± 1.3
Dyspnea (0–10)	8 ± 1.7
Leg effort (0–10)	5 ± 1.5

Abbreviations: HR: heart rate; SpO_2_: oxygen saturation; SBP: systolic blood pressure; DBP: diastolic blood pressure; LVEF: left ventricular ejection fraction

Data are expressed as n (%) unless otherwise stated

### Exercise duration and functional parameters

**Fig 2:**
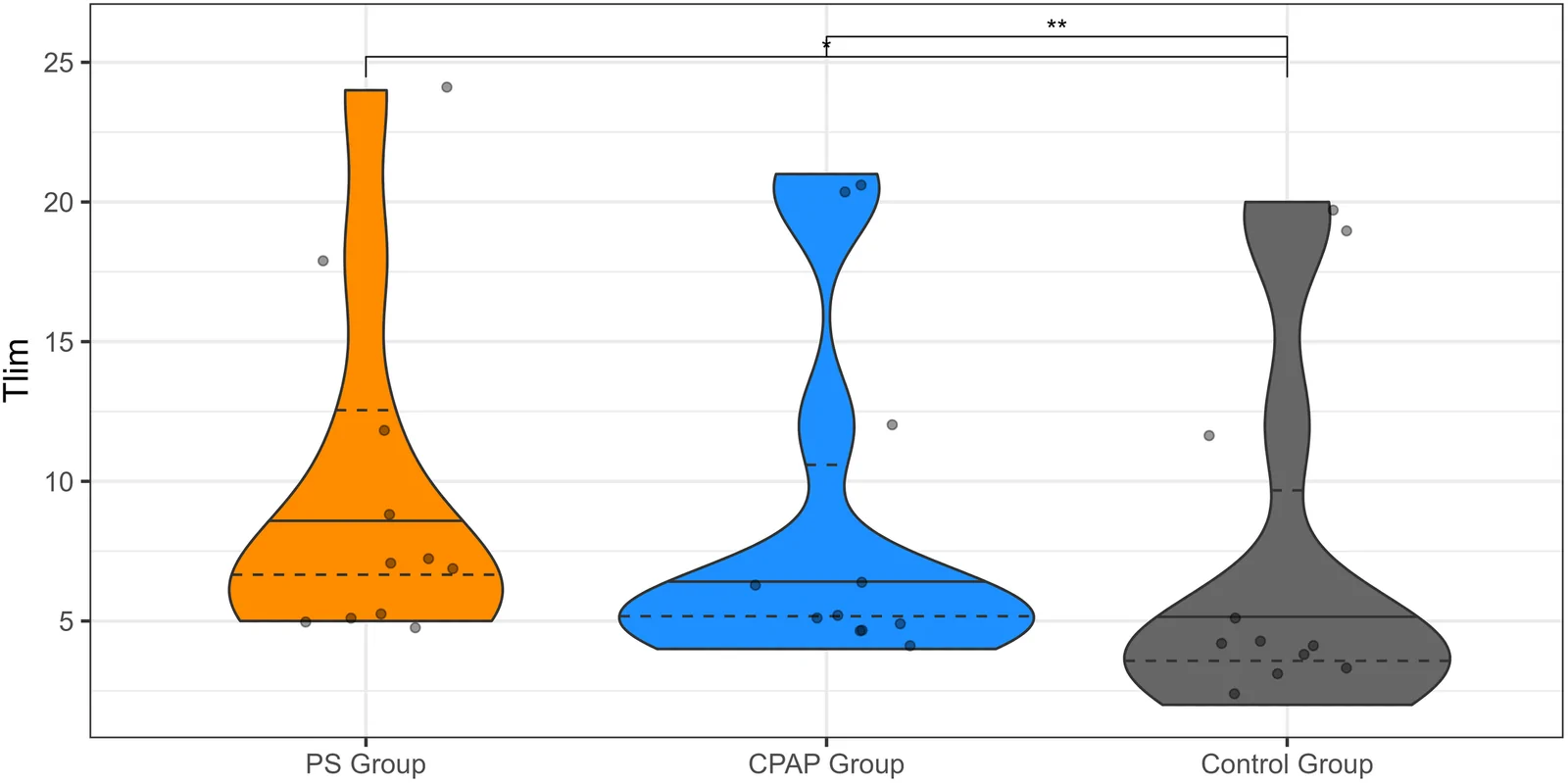
Violin plot representing the maximum time achieved (Tlim) during the test for each group. The median is indicated by the horizontal black line within each plot and the first and third quartiles by the dashed lines. The Friedman test revealed significant differences among groups (χ²(2) = 12.18, p = 0.0023, n = 11). Pairwise comparisons using the Wilcoxon test showed a statistically significant difference between Group 1 (PS) and Control Group 3 (p < 0.01). PS, pressure support; CPAP, continuous positive airway pressure.

The maximum Tlim differed significantly between groups (p = 0.002 and a large effect size, Kendall’s W = 0.554). The PS group achieved the longest duration (9.45 ± 1.88 minutes), followed by the CPAP group (8.55 ± 1.89 minutes), while the control group exhibited the shortest exercise time (7.27 ± 1.99 minutes). More information in Fig 2.

**Fig 3:**
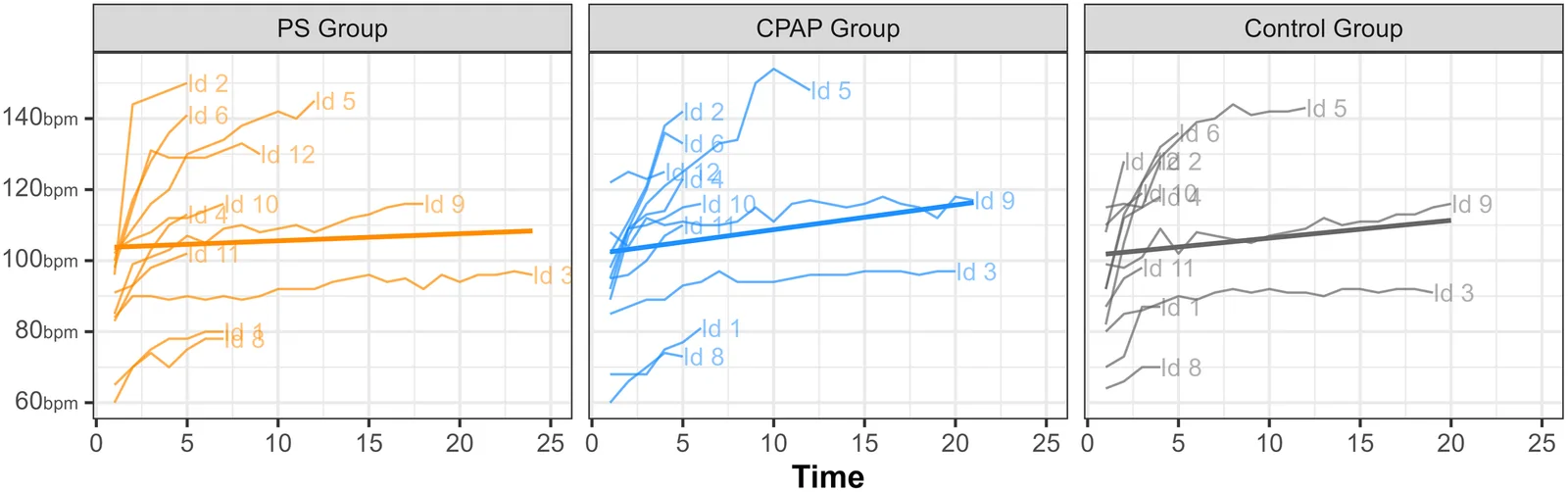
Longitudinal profiles of heart rate for each subject in each group.

No significant differences were observed in final heart rate (HR) (p = 0.946) or oxygen saturation (SpO₂) (p = 0.097) at the end of the tests. Similarly, subjective measures—including Borg scale scores and perceived fatigue—showed no significant differences between groups at test completion (p = 0.103 and p = 0.223, respectively). More information in Fig 3.

Peak HR values were consistent across groups (p = 0.965). The PS group reported a median peak HR of 98 beats/min (range: 77.5–117), the CPAP group reached 100 beats/min (range: 80–112), and the control group recorded 100 beats/min (range: 74.5–113).

Similarly, SpO₂ values showed no significant differences between groups. Initial SpO₂ medians ranged from 96.5% to 97.1%, while final values varied between 95.91% and 97.18% (p = 0.965 and p = 0.546, respectively). A summary of these results is presented in Table 2.

**Table 2:** Comparison of the three tests at the end of the protocol.

	PS group	CPAP group	Control group	F/χ^2^	df	p-value	η_p_^2^/W
Tlim (min)	9.5 ± 1.9	8.6 ± 1.9	7.3 ± 2.0	12.2	2	0.002	0.554
HR (bpm)	115.2 ± 7.5	115 ± 7.1	112.2 ± 6.9	0.1	2,30	0.946	0.004
SpO_2_ (%)	96.4 ± 0.6	96.3 ± 0.9	94.3 ± 1.2	4.7	2	0.097	0.212
Dyspnea (Borg scale 0–10)	8.1 ± 0.4	8.6 ± 0.4	7.3 ± 0.5	2.5	2,30	0.103	0.141
Fatigue (Borg scale 0–10)	6.3 ± 1.0	7.3 ± 0.8	6.6 ± 0.6	3	2	0.223	0.136

Tlim, time limit to exhaustion; HR, heart rate; SpO_2_, oxygen saturation; PS, pressure support; CPAP, continuous positive airway pressure; df, degrees of freedom

Values are reported as means, standard error of the mean, the test statistic F or χ^2^ p-values and the effect size η_p_^2^ or Kendall’s W from repeated measures ANOVA or Friedman test

### Parameters at specific time points

Iso-time analysis at minute 2 showed no significant differences in HR (p = 0.983) or SpO_2_ (p = 0.850) among the groups. However, fatigue perception was significantly higher in the no-support group (3.45 ± 0.61) compared to both NIV support groups (2 ± 0.27; p = 0.013 and a moderate effect size, Kendall’s W = 0.481). More information in Table 3. Percentile analysis at 25%, 50%, and 75% of exercise time revealed no significant differences between groups for HR, SpO_2_, Borg scale, or fatigue (all p > 0.05).

**Table 3:** Comparison of internal load measures at minute 2 (t = 2) across groups.

	PS group	CPAP group	Control group	F/χ^2^	Df	p-value	η_p_^2^/W
HR (bpm)	100.5 ± 6.44	99.2 ± 5.6	100.5 ± 5.8	0.017	2,30	0.983	0.001
SpO₂ (%)	97.2 ± 0.4	96.6 ± 0.6	95.9 ± 0.8	1.47	2	0.479	0.067
Borg scale (Dyspnea)	2.9 ± 0.4	3.2 ± 0.6	4.0 ± 0.6	1.05	2,30	0.364	0.067
Borg scale (Fatigue)	2.0 ± 0.3	2.0 ± 0.3	3.4 ± 0.6	8.67	2	0.013	0.481

HR, heart rate; SpO_2_, oxygen saturation; PS, pressure support; CPAP, continuous positive airway pressure: df, degrees of freedom

Values are reported as means, standard error of the mean, the test statistic F or χ^2^ p-values and the effect size η_p_^2^ or Kendall’s W from repeated measures ANOVA or Friedman test

### Reason for test termination and comfort

The primary reasons for test termination were consistent across groups, with 54.5% of participants stopping due to dyspnea and 45.5% due to fatigue.

Global comfort scores for the NIV support devices showed no statistically significant differences between groups, both pre- and post-exercise.

No adverse events related to the intervention were reported in any group. Additionally, no participants experienced significant side effects due to NIV use during the exercise protocol.

### Post hoc power analysis

Based on the observed effect sizes and sample variability, the statistical power was estimated at 24% for comparisons between the CPAP and control conditions, 74% for CPAP versus pressure support, and 99% for pressure support versus control. These findings suggest that while the comparison between pressure support and control was adequately powered, the study may have been underpowered to detect differences between CPAP and the other two conditions.

## Discussion

This study demonstrated that NIV, particularly in pressure support mode, significantly increased exercise duration in patients with HFrEF compared to no ventilatory support. Both the PS and CPAP groups achieved significantly longer Tlim than the control group, suggesting that both NIV modalities effectively improve exercise tolerance in this population.

### Mechanisms underlying the benefits of NIV during exercise

Most patients with HF experience dyspnoea and fatigue as two of the most significant and limiting symptoms, both of which are associated with a decline in functional capacity and quality of life [23]. The multifactorial origins of these symptoms include impaired respiratory mechanics, altered metaboreflex responses, and increased cardiac workload [24,25]. NIV during exercise offers several physiological benefits, including enhanced respiratory mechanics, improved oxygenation, and reduced cardiac load. These effects are mediated by improved muscle oxygenation during recovery [11] a more efficient metaboreflex response [12], and lower left ventricular transmural pressure [13], all of which contribute to delaying fatigue and optimizing performance.

To date, only two studies have evaluated the effectiveness of CPAP and PS during exercise in patients with HF. The studies by O’Donnell et al. [15], and Reis et al. [12], used a CWRT protocol at 75% of the maximal workload and reported significant results in favour of the NIV group regarding Tlim to exhaustion. Our findings demonstrated significant improvements in Tlim with both CPAP and PS modes compared to no NIV support, further supporting the efficacy of NIV during exercise in enhancing aerobic endurance.

### Comparisons with prior research

Our findings align with prior research highlighting the benefits of NIV in reducing respiratory muscle workload and improving functional capacity in patients with HF. For instance, studies by Borghi-Silva et al. [11] and Chermont et al. [26] demonstrated that NIV modalities like CPAP and proportional assist ventilation improved exercise tolerance by decreasing dyspnea and facilitating oxygen delivery during exercise.

The superior performance of the PS group could be explained by its ability to relieve the respiratory muscles, improve ventilatory efficiency, and redistribute blood flow to peripheral muscles during exercise [27–29]. Similar effects have been described in studies by Lima et al. [30] and O’Donnell et al. [15], who reported that reducing the respiratory workload through NIV improves peripheral muscle oxygenation and delays the onset of fatigue. In this regards, the application of NIV during exercise could possibly attenuate the inspiratory metaboreflex impact on physical performance, but this hypothesis requires further investigation [31].

Although our study found no statistically significant differences in HR trends between the groups (p-trend), the pattern observed in the PS group, with a flattening of HR during exercise, is consistent with findings from previous studies by Reis et al. [12] and O’Donnell et al. [15] These studies reported significant reductions in HR with the use of NIV during constant work rate exercise, suggesting that NIV may help stabilize cardiovascular responses during physical exertion. Moreover, the observed reduction in fatigue perception at minute two in our cohort underscores the potential of NIV to attenuate early exertional symptoms, which can limit exercise participation in HF patients.

### Clinical implications and feasibility of NIV in rehabilitation

The findings support the addition of NIV, particularly the PS mode, during exercise for HFrEF patients to enhance exercise tolerance and potentially improve adherence to physical activity regimens. The prolonged Tlim in the PS group suggests that this mode of ventilation might be particularly beneficial for patients with advanced disease who experience significant dyspnea and fatigue during exercise. Additionally, the similar subjective comfort scores across all conditions indicate that both CPAP and PS are well-tolerated modalities, making them feasible options for broader implementation in clinical settings.

### Limitations and future directions

A key limitation of this study is that oxygen consumption (VO_2_) was not directly evaluated in this study. Although VO_2_ analysis is a valuable parameter for assessing cardiorespiratory fitness, it is not compatible with the simultaneous use of NIV due to interference with gas exchange measurements and mask integrity during ventilation. As a result, we prioritized the use of reliable and practical measures such as CWRT and perceived exertion, which are well-established markers of exercise tolerance in clinical settings [32].

Additionally, our study focused exclusively on patients with HFrEF. Therefore, the findings cannot be generalized to individuals with preserved or mildly reduced ejection fraction, who may have different pathophysiological responses to exercise and non-invasive ventilation. Future studies should investigate whether similar effects are observed in these subpopulations.

Another limitation is the small sample size. Although the original sample size calculation estimated that 12 participants were needed, one subject was excluded due to intolerance to NIV, resulting in a final sample of 11. Despite this, the crossover design helped to maintain statistical power by allowing each participant to serve as their own control. Nevertheless, the reduced number of participants may limit the generalizability of the results. The post hoc power analysis provides additional context for interpreting the results. While the improvement in exercise time with pressure support was statistically significant and associated with a high power (99% vs. control), the lower power (24%) for the CPAP vs. control comparison suggests that this study may not have been sufficiently powered to detect smaller differences between these conditions. This limitation should be considered when interpreting the non-significant findings, particularly those between CPAP and control, and highlights the need for further research with larger sample sizes to fully characterize the comparative effects of different NIV modalities.

Finally, the sample showed imbalance in certain clinical characteristics, such as sex distribution (73% male) and heart failure etiology (81.8% ischemic). While the crossover design minimizes the impact of inter-individual variability, these imbalances may limit the applicability of findings to more diverse heart failure populations.

### Strengths of the study

A key strength of this study is the direct comparison of two modes of NIV during exercise, which differentiates it from most previous research. According to the literature reviewed, numerous studies have employed NIV prior to assessments [26,30,33–35], limiting its applicability in the assessment of dynamic responses during physical exertion. This innovative approach allowed for a more accurate analysis of the real-time effects of NIV, providing data directly applicable to clinical and rehabilitation settings. Additionally, the use of CWRT represents another significant strength of the study design. This protocol is highly sensitive for evaluating aerobic endurance and enables the detection of small performance changes following interventions, making it a key tool for assessing the impact of therapeutic strategies such as NIV [11,15,33]. Unlike incremental tests, CWRT provides a stable and reproducible assessment of exercise tolerance by focusing on endurance capacity under controlled conditions. Finally, an additional strength of this study is its design, which allows each participant to serve as their own control. This approach minimizes inter-individual variability and enhances the reliability of the comparisons between the PS, CPAP, and no-support conditions. By controlling for individual baseline differences, the crossover design provides robust evidence that the observed improvements in Tlim are directly attributable to the use of NIV, rather than being influenced by other confounding factors.

Future research should explore the effects of combining NIV with other interventions, such as resistance training [34,35] or inspiratory muscle training, to provide a more comprehensive approach to optimizing exercise capacity in this patient population.

## Conclusion

This study demonstrates that NIV, particularly with PS mode, significantly increased maximum exercise time compared to CPAP and no support mode. Additionally, NIV support was associated with a reduction in perceived fatigue at iso-time points, highlighting its potential role in improving exercise tolerance in patients with heart failure. These findings support the integration of NIV as an adjunctive therapy to enhance functional capacity during exercise in this population. Further studies are needed to optimize its application and determine the most effective modes of NIV.

## Supporting information

S1 Data Minimal dataset used to generate the tables and figures in the study. (XLSX)

S1 Protocol (PDF)

S1 CONSORT Checklist Completed CONSORT checklist detailing adherence to reporting standards for randomized crossover trials. (DOCX)
