# Supplementary material for: Is non-invasive ventilation effective in improving the exercise capacity in patients with cardiac heart failure?: A randomised crossover trial
Source: PLoS One. 2025 Jul 7;20(7):e0327399. doi: 10.1371/journal.pone.0327399 (PMC12233218; doi:10.1371/journal.pone.0327399)
Supplement: Supplementary file 3 [file pone.0327399.s002.pdf]

RESEARCH AND DEVELOPMENT PROJECT

EVALUATION FORM
YEAR 2021

1. PROJECT IDENTIFICATION

1.1 PROJECT TITLE:

"Evaluation of the Use of Non-Invasive Ventilation (NIV) in the Exercise Capacity of Patients with Heart Failure"

PROJECT CODE: (To be completed by SIT)

1.2 PROJECT DIRECTOR

Last Name and First Name: Intelangelo Leonardo
ID Type and Number: DNI
Home Address:
Phone:
E-mail:

1.3 TYPE OF RESEARCH:

Experimental Study

1.4 DISCIPLINE AND SPECIALTY: (Select one option)

Specialization in Kinesiology and Cardiorespiratory Physiotherapy

☐ **Rosario** ☐ Santa Fe ☐ Venado Tuerto ☐ Marco Juárez

1.7 PRIORITY AREA ADDRESSED:

Cardiorespiratory Rehabilitation

1.8 IS THIS A CONTINUATION OF A PREVIOUS PROJECT? (If yes, provide a brief explanation)

No

1.9 SUMMARY: (maximum 500 words; structured as: Background, General Objective, Methodology, Keywords)

Patients with heart failure (HF) have reduced exercise tolerance as a primary consequence of the disease. (1) This reduced tolerance is due not only to cardiac conditions but also to dysfunction of respiratory and peripheral muscles. (1) Several factors, such as chronic hypoxia, oxidative stress, nutritional depletion, peripheral muscle disuse, medication effects, and sympathetic-vagal imbalance, are important contributors. (2)

In this scenario, the use of non-invasive ventilation (NIV) has emerged as an adjunct to cardiac rehabilitation in an attempt to improve patients' functional capacity. NIV reduces respiratory workload, improves oxygenation, and increases pulmonary compliance, which is associated with improved ejection volume due to increased intrathoracic pressure. In this context, non-invasive ventilation (NIV) could help enhance physical performance.

The isolated use of NIV has been recommended to improve pulmonary, cardiac, and peripheral muscle functions, while the use of NIV in combination with physical exercise as a complementary strategy to increase exercise tolerance has been widely investigated in pulmonary rehabilitation programs. However, there is limited evidence in the field of cardiac rehabilitation. Three studies have examined the benefits of NIV during exercise in patients with stable advanced HF. (6)(9)(15)

Our objective is to evaluate whether the use of the Pressure Support (PS) mode of NIV during exercise improves aerobic exercise tolerance in patients with HF. To achieve this, the proposed study will follow a crossover design and will be conducted at the Cardiovascular Rehabilitation Service of the Cardiovascular Institute of Rosario (ICR).

Keywords: Non-Invasive Ventilation; CPAP; Bilevel; Heart Failure; Cardiac Rehabilitation; Physical Exercise.

2. INVESTIGATION PLAN

2.1 PROJECT DESCRIPTION (maximum two pages)

Heart failure (HF) is a complex clinical syndrome that can result from any structural or functional cardiac disorder that impairs the ventricle's ability to fill or eject blood. The cardinal manifestations are dyspnea and fatigue, which can limit exercise tolerance, as well as fluid retention, which may lead to pulmonary congestion and peripheral edema. Both abnormalities can impair functional capacity and quality of life in affected individuals, though they do not necessarily dominate the clinical presentation simultaneously. (3)

Currently, some ventilatory support resources have begun to be used in cardiac rehabilitation programs to increase exercise tolerance. The use of non-invasive ventilation (NIV) is emerging as a therapeutic option for groups in which it is believed to improve ventilatory mechanics and oxygenation, optimizing cardiorespiratory function and thereby enhancing exercise capacity.

Traditionally, NIV has been used in cases of respiratory failure and in HF patients to reverse pulmonary edema and respiratory insufficiency. Research on the use of NIV and its effects on exercise tolerance has only recently begun, but there are controversies regarding its effectiveness and clinical application. (4)

Few studies have assessed the response of this therapeutic approach in improving physical exercise performance in HF patients. Additionally, some studies have only evaluated the impact of NIV use at rest on exercise tolerance, rather than the combined effect of NIV and physical exercise. Understanding the efficacy of NIV as a supportive therapeutic approach in prescribing exercise for HF patients is particularly important in cardiovascular therapy. These patients have reduced exercise tolerance, and improving peripheral muscle function is a primary goal of physiotherapy interventions. (5)

Therefore, due to the limited evidence on the use of NIV during aerobic effort, we have designed this randomized crossover study to evaluate whether the use of NIV during exercise improves aerobic exercise tolerance in HF patients. Additionally, subjective perception of dyspnea and fatigue will be collected and analyzed during the tests, along with oxygen saturation, heart rate, respiratory rate, and blood pressure values.

2.2 CURRENT STATE OF KNOWLEDGE ON THE TOPIC

A bibliographic search was conducted in the following electronic databases: MEDLINE, LILACS, and the Spanish Journal of Cardiology search engine. The search was performed in May 2020. The following MeSH terms were used: “Heart Failure”, “non-invasive ventilation”, “bilevel”, “exercise”. The words were combined to perform the search using the Boolean operator AND and restricting the research to these terms within the title and abstract fields of each database. The inclusion

criteria were randomized studies, with participants diagnosed with HF without chronic obstructive disease or other coexisting pulmonary diseases, studies evaluating the impact of NIV on exercise tolerance in individuals with HF. The publications had to be in English or Spanish. The exclusion criteria were non-randomized studies, studies with participants with HF and chronic obstructive pulmonary disease or other coexisting pulmonary diseases, studies applying NIV without evaluating exercise tolerance.

A selection of the published articles was carried out in four phases. In the first phase, the titles of all published articles related to the combination of search terms were read, and those that did not meet the inclusion criteria of this review were excluded. In the second phase, the titles from the different databases were read, and those that were duplicated were recorded. In the third phase, the abstracts of the articles selected in the first phase were read, and those that did not meet the inclusion criteria of this review were excluded. In the fourth and final phase, the remaining articles were read in detail and analyzed to conclude which would finally be selected for inclusion in the review.

Finally, by combining the MeSH terms and Boolean operators, a total of 51 articles were found in the databases. Out of the total number of articles found, a total of ten articles were selected (6)(7)(8)(9)(10)(11)(12)(13)(14)(15).

2.2.2 Background

As indicated in the previous section, 10 articles evaluated the impact of NIV on exercise tolerance in individuals with HF. However, considering the timing of NIV application, six authors applied NIV for 30 minutes before exercise (7)(8)(10)(11)(13)(14), three applied it during exercise (6)(9)(15), and only one author applied nocturnal NIV for at least 6 hours over approximately 8 months (12). Therefore, if we consider the timing of use, only three articles evaluated the use of NIV during exercise (6)(9)(15).

Within NIV, we have different ventilatory modes: continuous positive airway pressure (CPAP), pressure support (PS), and Bilevel or Bipap. In CPAP mode, the patient breathes spontaneously at a pressure level higher than atmospheric pressure, which allows for the generation of a positive pressure gradient within the inspiration-expiration cycle. The effect of positive pressure on both phases of the respiratory cycle increases the mean airway pressure, consequently increasing the functional residual capacity (FRC) and reducing respiratory effort. The PS modality is a form of assisted ventilation in which the patient triggers the ventilator on a breath-by-breath basis. The operator determines the pressure level to be supplied, which is maintained throughout the patient's entire spontaneous inspiratory cycle. Finally, Bilevel support is a ventilatory mode in which the cycle occurs between an inspiratory positive airway pressure (IPAP) and a final expiratory positive airway pressure (EPAP/PEEP). In this case, there is ventilatory support and increased oxygenation (16).

Therefore, considering the previously described ventilatory modes, one article used CPAP (9), and the remaining two used PS (6)(15).

O'Donnell et al. (6) were among the first to question whether the use of ventilatory support could have an impact on exercise tolerance in patients with HF. In response to their concerns, they developed a clinical trial in which they applied PS and also CPAP in a group of 12 participants diagnosed with HF. Their findings showed that patients who performed exercises using NIV experienced an increase of 2.8 ± 0.8 minutes in exercise duration with PS and an increase of 1.4 ± 0.7 minutes with CPAP compared to those who did not use ventilatory support ($p<0.01$). Additionally, at a standardized time point near the end of the tests, PS and CPAP reduced respiratory effort per minute by 39 ± 8% and 25 ± 4%, respectively ($p<0.01$).

Borghi-Silva A et al. (15) conducted a study with 9 patients with HF, in which, after an incremental cardiopulmonary exercise test using a ramp protocol on a cycle ergometer, patients performed, on separate days, a high-intensity constant-load test (CLT) at 70-80% of their maximum workload. Patients received PS or placebo ventilation, and the effects of respiratory muscle unloading and its relationship to leg muscle oxygenation and blood volume during high-intensity exercise were analyzed. Significant improvements in exercise tolerance (Tlim) were observed with PS (290 s vs. 366 s - $P<0.01$). Blood lactate, perceived leg fatigue/Tlim, and dyspnea/Tlim were lower with PS compared to placebo ventilation ($P<0.05$). The authors concluded that respiratory muscle unloading had beneficial effects on muscle oxygenation status and blood volume during exercise. These data suggest that blood flow was redistributed from respiratory muscles to locomotor muscles during tests with PS.

Finally, Reis et al. (9) conducted a study in which 7 men with HF (62 ± 8 years) and a left ventricular ejection fraction of 41 ± 8% underwent a symptom-limited incremental exercise test (IT) on a cycle ergometer. On separate days, patients were randomly selected to perform four CLTs until maximum tolerance with and without CPAP under the following conditions: first at 50% of the maximum workload from the IT, then at 75% of the peak workload from the IT. At rest and under these conditions, heart rate (HR) and other variables were recorded. Regarding tolerated time in the CLT, a statistically significant difference was observed in favor of CPAP use vs. spontaneous breathing during the CLT at 75% of IT ($p<0.05$). Additionally, HR values were higher with CPAP compared to spontaneous breathing at 75% intensity ($p<0.05$). The results of this study are consistent with the previously mentioned publications.

2.3 OBJECTIVES OF THE PROJECT

2.3.1 Main Objective

- Compare the maximum tolerated time in seconds in a CLT with the use of PS, CPAP, and without the use of ventilatory support in patients with non-decompensated HF (CLT: PS vs CPAP vs GSSV).

2.3.2 Specific Objectives

- Quantify and compare the subjective sensation of dyspnea in the different exercise tests (during rest, isochronal time, and end of effort - CLT: PS vs CPAP vs GSSV).
- Determine and compare SpO2 in the different exercise tests (during rest, isochronal time, and end of effort - CLT: PS vs CPAP vs GSSV).
- Study and compare other physiological or effort-related variables (heart rate, respiratory rate, blood pressure, and lower limb fatigue) in the different exercise tests (during rest, isochronal time, and end of effort - CLT: PS vs CPAP vs GSSV).
- Determine the comfort of using both NIV modes during the tests. At rest and end of effort (CLT: PS vs CPAP).

2.3.3 Hypothesis

- The use of PS will improve exercise tolerance compared to the use of CPAP and the non-use of ventilatory support in HF patients during different exercise tests (CLT).

2.4 METHODS

2.4.1 Design

- Crossover experimental study to be developed in the Rehabilitation Service of the ICR in Rosario.

2.4.2 Study Population

A sample of patients with compensated HF will be studied, whether or not they are within the cardiac rehabilitation program at the Cardiovascular Institute of Rosario (ICR).

2.4.3 Inclusion Criteria

● Ejection fraction demonstrated by echocardiography less than 40% (less than 6 months)

● New York Heart Association (NYHA) Class I – III

● Clinical stability of their disease, defined as four weeks before the start of the study without hospitalization requirements.

● Signed informed consent (see annex).

● Over 18 years old.

2.4.4 Exclusion Criteria

● Patients with neuromuscular or osteoarticular disorders that contraindicate the evaluations.

● Chronic obstructive pulmonary disease (COPD), unstable angina, or significant arrhythmias, myocardial infarction in the last 3 months, primary valvular disease, and anemia.

● Smokers or former smokers of less than one year.

● Patients with cognitive impairment that prevents proper understanding of the evaluations.

2.4.5 Study Development

The study will be carried out in 4 visits separated by a minimum period of 24-48 hours to allow physical recovery, and in the case of patients within the CR program, before the day's training session, ensuring adequate rest between visits.

Four tests will be performed on a treadmill, one incremental test (IT) to determine the patient's maximum aerobic speed (MAS), defined as the maximum speed reached in km/h during the entire test stage, and subsequently, three constant-load tests (CLT), where the primary variable evaluated will be the time limit (Tlim). Of the three CLTs, one will be performed breathing ambient air without ventilatory support (GSSV), another with PS, and the other using CPAP. The randomization of the test order (PS vs CPAP vs GSSV) will be conducted through the website "randomization.com".

The NIV tests will be conducted with an oronasal interface, with a support pressure of 5 cmH_2O (10 IPAP and 5 EPAP) for PS mode and a pressure of 5 cmH_2O for CPAP mode. These pressures have been established based on literature review and the research team's experience. The GSSV tests will be conducted without any system.

We do not expect patients to experience oxygen desaturation as verified by pulse oximetry during the tests. If a patient desaturates below 85% during the IT, the test will be stopped, and a Venturi mask with an oxygen flow that maintains saturation above 88% will be applied. In this case, the patient will perform the three subsequent CLTs with the same FiO_2 used during the IT. For patients who do not require oxygen during the IT, the tests will be conducted without supplemental oxygen.

Before each test, for the NIV group, the system will be set up, and the patient and system will be allowed 5 minutes to stabilize. Then, SpO_2, HR, RR, BP, overall method comfort, and symptoms of dyspnea and lower limb fatigue will be recorded, and the corresponding test will begin.

Due to the current SARS-CoV-2 pandemic, the evaluation team will wear personal protective equipment, including N95 masks and face shields, ensuring proper ventilation of the spaces and maintaining distance from other patients. All equipment used will be either new or properly sterilized beforehand.

Visit 1: Inclusion in the Study and IT

Identification of patients meeting the selection criteria for protocol enrollment.

Explanation of the study to the patient and signing of the informed consent.

Performance of the first and only IT on a treadmill to assess their maximum exercise capacity (MAS in km/h). The test will start at a speed of 3 km/h and increase by 0.5 km/h every minute. At each speed change, SpO2, HR, and symptoms of dyspnea and lower limb fatigue will be recorded.

Randomization for the order of CLTs in visits 2, 3, and 4.

The test will be stopped at the patient's request and/or for clinical and electrocardiographic reasons according to the Spanish Society of Cardiology's guidelines (17).

At the end of the test, the maximum aerobic speed (MAS), corresponding to the last completed minute's speed, will be recorded along with SpO2, HR, RR, BP, dyspnea symptoms, fatigue, and overall method comfort.

Visit 2: First Constant Load Test (CLT)

The CLT will be performed according to the randomization: CLT-PS, CLT-CPAP, or CLT-GSSV.

The CLT will include a 5-minute warm-up at 50% of the MAS, followed by treadmill walking at a constant speed corresponding to 80% of the MAS. A 50% warm-up level was chosen based on two prior studies that selected evaluation percentages between 70-85% (7)(13). SpO2, HR, and symptoms of dyspnea and fatigue will be recorded every minute.

The test will be stopped based on the same criteria as the IT or if a duration of 15 minutes is reached, excluding the warm-up time. If the patient reaches 15 minutes without exhaustion, the workload will be increased by 5%, and the test will be repeated.

Visit 3: Second Constant Load Test (CLT)

The second CLT will be performed in the order assigned by randomization: CLT-PS, CLT-CPAP, or CLT-GSSV, following the instructions described in visit 2.

Visit 4: Third and Final Constant Load Test (CLT)

The third CLT will be performed in the order assigned by randomization: CLT-PS, CLT-CPAP, or CLT-GSSV, following the instructions described in visit 2.

2.4.6 Adverse Events

a. During exercise capacity tests: Adhering to absolute and relative contraindications for exercise testing, ITs and CLTs are safe tests. Adverse events associated with ITs are rare (0.16%), and no fatal events have been reported. CLTs have not been associated with adverse events in the literature (18). Monitoring of HR and SpO2 during the test is recommended, and testing should stop if SpO2 falls below 85%.

b. Use of NIV: No major complications are expected. Discomfort due to pressure, high flow rates, discomfort, or skin marks from the interfaces may occur.

2.4.7 Exclusion Criteria

The subject may be excluded from the study if:

● An event occurs during any test requiring forced termination (19).

● An acute HF exacerbation requires hospitalization or medication changes during the study.

● The patient is absent or delays visits beyond 7 days.

● The patient refuses to continue participation.

2.4.8 Sample Calculation

For the sample calculation, the maximum time in the CLT was chosen as the primary study variable because it is a sensitive measure for detecting changes after interventions (both pharmacological and non-pharmacological) and is widely used in clinical trials.

A minimum detectable difference of 100 seconds was determined based on previous literature. The sample size was calculated based on the following assumptions derived from the results of a previous study (6): 1- Paired t-test analysis for repeated measures; 2- Difference to be detected of 1.4 minutes between PS, CPAP, and no ventilatory support trials; 3- G-power determination of 0.80; and an alpha value of 0.05. A sample of 12 patients is needed.

2.4.9 Measurement Variables

● **Sample Categorization Variables:**

1. Age: years (dd/mm/yyyy ENTRY) – (dd/mm/yyyy BIRTH)
2. Gender: M/F
3. Height: cm
4. Actual weight (Pr): kg

5. Theoretical weight (Pt): Male: 50 + 0.91 (height in cm – 152.4), Female: 45.5 + 0.91 (height in cm – 152.4)
6. Body mass index (BMI): Kg/m^2
7. Chronic home oxygen therapy (CHOT): YES/NO
8. Ejection fraction (EF): %

● **Study Variables:**

- Maximum aerobic speed (MAS) of IT: in Km/h
- Time limit (Tlim) of CLT: in seconds
- Baseline, isochronal, and final oxygen saturation (SpO_2): in %
- Baseline, isochronal, and final dyspnea: Modified Borg Scale (0-10)
- Baseline, isochronal, and final heart rate (HR): beats per minute
- Baseline and final respiratory rate (RR): breaths per minute
- Baseline and final blood pressure (BP): mmHg
- Baseline, isochronal, and final lower limb fatigue: Modified Borg Scale (0-10)
- Overall comfort sensation of the oxygen administration method, baseline and final: Visual Analog Scale (VAS) 0-10

2.4.10 Materials

- Treadmill (KIP Machines - KR RS-320)
- Non-invasive ventilation (Philips Respironics)
- Pulse oximeter (Masimo SET Radical-7)
- Manual sphygmomanometer (HAND)
- Modified Borg Scale (see annex)
- Visual Analog Scale (see annex)
- Stopwatch
- Data collection forms (see annex)

2.4.11 Ethical Aspects

The project will adhere to the Declarations of the World Medical Association of Helsinki.

The different evaluation procedures described in this project have been previously clearly defined by multiple authors, and all are non-invasive and harmless, posing no risk of adverse health effects.

The researchers have experience in managing adults with cardiopulmonary conditions and in using evaluation instruments both in clinical practice and research.

To ensure the protection of participants, all relevant aspects of the safety of the evaluation procedures will be duly noted in the informed consent (see Annexes), and all personal data and evaluation-derived data will be kept confidential. It will be clearly stated in the consent that the use of the results for scientific publication purposes will only be carried out with the consent and authorization of the participants.

The participation of all individuals will be free and voluntary. The recruitment will be conducted through oral and printed dissemination at the Cardiovascular Institute of Rosario, as well as through direct referral by the institute's cardiologists. Finally, all participants will have the opportunity to contact the project researchers via phone or email, both before and after their evaluation and intervention.

Beforehand, the researchers will present and explain the importance of informed consent. Participants will be required to read it carefully and sign it if they agree. The same researchers will be responsible for providing additional information if requested. The study may be suspended at any time if the patient wishes.

The project has been approved by the teaching and research committee of the Cardiovascular Institute of Rosario for implementation at the institution (approval letter attached).

2.5 REFERENCIAS BIBLIOGRÁFICAS

1. Dempsey JA, Romer L, Rodman J, Miller J, Smith C. Consequences of exercise-induced respiratory muscle work. Respir Physiol Neurobiol. 2006;151:242-50.
2. Gosker HR, Wouters EF, van der Vusse GJ, Schols AM. Skeletal muscle dysfunction in chronic obstructive pulmonary disease and chronic heart failure: underlying mechanisms and therapy perspectives. Am J Clin Nutr. 2000;71:1033-47.
3. Thierer J. Insuficiencia cardíaca : evidencia, experiencia y opinión . 1st ed. Vol. 3. Buenos Aires; 2013. 54–67 p.
4. Bittencourt et al. Non-Invasive Ventilation in Patients with Heart Failure: A Systematic Review and Meta-Analysis. Arq Bras Cardiol. 2017; 108(2):161-16
5. Dempsey JA, Romer L, Rodman J, Miller J, Smith C. Consequences of exercise-induced respiratory muscle work. Respir Physiol Neurobiol. 2006;151:242-50.
6. O'Donnell DE, D'Arsigny C, Raj S, Abdollah H, Webb KA. Ventilatory assistance improves exercise endurance in stable congestive heart failure. Am J Respir Crit Care Med. 1999;160(6):1804–11.
7. Chermont S, Quintão MMP, Mesquita ET, Rocha NN, Nóbrega ACL. Noninvasive ventilation with continuous positive airway pressure acutely improves 6-minute walk distance in chronic heart failure. J Cardiopulm Rehabil Prev. 2009;29(1):44–8.
8. Da Silva E, Cruz CG, Santos FC, Gomes-neto M, Souza H, José F, et al. Artículo Original Soporte Ventilatorio en la Capacidad Funcional de Pacientes con. Arq Bras Cardiol. 2011;96(3):227–32.
9. Reis H V., Borghi-Silva A, Catai AM, Reis MS. Impact of CPAP on physical exercise tolerance and sympathetic-vagal balance in patients with chronic heart failure. Brazilian J Phys Ther. 2014;18(3):218–27.

10. Bussoni MF, Guirado GN, Matsubara LS, Roscani MG, Polegato BF, Minamoto ST, et al. Diastolic function and functional capacity after a single session of continuous positive airway pressure in patients with compensated heart failure. Clinics. 2014;69(5):354–9.
11. Gomes Neto M, Duarte LFG, Rodrigues E de S, Bittencourt HS, dos Santos NG, David BC, et al. Effects of noninvasive ventilation with bilevel positive airway pressure on exercise tolerance and dyspnea in heart failure patients. Hell J Cardiol. 2018;59(6):317–20.
12. Steiner S, Schannwell CM, Hennersdorf M, Strauer BE. Effects of continuous positive airway pressure on exercise capacity in 20 chronic heart failure. Soc Sci. 2007;665–72.
13. Wittmer VL, Simoes GMS, Sogame LCM, Vasquez EC. Effects of continuous positive airway pressure on pulmonary function and exercise tolerance in patients with congestive heart failure. Chest. 2006;130(1):157–63.
14. Carvalho L de A, Brandão DC, Campos SL, Vidal TM de S, Remígio MI, Martins SM, et al. Noninvasive Ventilation Before Maximum Exercise Test Increases Exercise Tolerance in Subjects With Heart Failure: A Crossover Study. Arch Phys Med Rehabil. 2017;98(5):849–55.
15. Borghi-Silva A, Carrascosa C, Oliveira CC, Barroco AC, Berton DC, Vilaça D, et al. Effects of respiratory muscle unloading on leg muscle oxygenation and blood volume during high-intensity exercise in chronic heart failure. Am J Physiol - Hear Circ Physiol. 2008;294(6):2465–72.
16. Ferrero R Gerardo. XII. Modos ventilatorios en ventilación no invasiva. Rev. chil. enferm. respir. 2008; 24(3): 240-250.
17. Fernando Arós et al. Guías de práctica clínica de la Sociedad Española de Cardiología en pruebas de esfuerzo. Rev Esp Cardiol. 2000;53:1063-94 - Vol. 53 Núm.08

18. Luis Puente-Maestu, Paolo Palange, Richard Casaburi, Pierantonio Laveneziana, François Maltais, J. Alberto Neder, et al. Use of exercise testing in the evaluation of interventional efficacy: an official ERS statement. Eur Respir J 2016; 47: 429–460.
19. ERS Task Force, Palange P, Ward SA, Carlsen KH, Casaburi R, Gallagher CG, Gosselink R, et al. Recommendations on the use of exercise testing in clinical practice. Eur Respir J. 2007 Jan;29(1):185-209

ANNEX 1

Informed Consent

"Evaluation of the Use of Non-Invasive Ventilation (NIV) in the Exercise Capacity of Patients with Heart Failure"

You are invited to participate in this research study entitled "Evaluation of the Use of Non-Invasive Ventilation (NIV) in the Exercise Capacity of Patients with Heart Failure", which is being conducted at the Cardiovascular Institute of Rosario to assess aerobic capacity. You have been invited to participate because you have been diagnosed with Heart Failure and belong to the group of patients required for this research study.

We kindly ask you to carefully read this document to understand what the research study entails and what your participation involves.

By signing this document, you confirm that the study has been explained to you and that you agree to participate. It is essential that you understand all details before making a decision about your participation.

Introduction

Heart Failure (HF) causes, among other things, shortness of breath and physical deconditioning in patients. Muscle weakness leads to a vicious cycle in which shortness of breath results in a sedentary lifestyle, which in turn leads to further weakness and even more inactivity. Cardiac Rehabilitation breaks this vicious cycle of immobility and breathlessness and consists of a personalized physical training program.

To properly design a rehabilitation plan tailored to each patient's specific needs, initial and periodic exercise capacity assessments must be performed. The training program will be based on the results of these assessments. Just as medications have a recommended dose, exercise training also has a "recommended dose" based on the assessment results. There are multiple evaluation methods depending on the specific exercise capacity being assessed.

For this reason, we invite you to participate in this study, where we will conduct four (4) aerobic capacity evaluations while walking on a treadmill. Our goal is to verify whether the system we will use during two of the tests can assist in exercise performance.

The system we will use and compare is called Non-Invasive Ventilation (NIV). Two of the four tests you will perform during the study will be conducted using this system. Non-Invasive Ventilation will deliver an airflow during both inhalation and exhalation.

Study Objective

To compare and verify whether there are differences in the results of aerobic capacity assessments, specifically in the duration you can walk at a constant speed across the three tests (two with a mask and one without any support). Additionally, we will assess your comfort and/or ease while using the NIV system during two of the three tests.

Details of Participation in the Study

Your participation in the study will require four separate days, with at least 24-48 hours of rest between each study session. Each evaluation session should not exceed 30 minutes. Below is a brief outline of the study schedule:

Day 1: We will conduct the first and only incremental load test. In this assessment, you will be asked to walk on a treadmill with a gradually increasing speed until you reach your maximum capacity.

Day 2: You will perform a constant load test, which means you will walk at a fixed speed for the duration of the test.

Day 3: You will perform a second constant load test, similar to the test on Day 2, but this time with NIV.

Day 4: You will perform a third and final constant load test, identical to those on Days 2 and 3, also with NIV.

The sequence of tests described above is just an example, as the order of the tests may be randomized.

We do not anticipate major complications during the tests, whether from walking on a treadmill or using the non-invasive ventilation mask. The discomforts that may arise are similar to those you might experience in daily life, such as leg fatigue, shortness of breath, discomfort from wearing the mask or nasal cannula, among others.

The necessary research data will be collected through interviews with volunteers and information from Electronic Medical Records, and will be handled exclusively by the researchers in a confidential manner to protect the identity and privacy of patients.

Your participation in this study is voluntary and free of charge, and there are no financial benefits for participating. Your participation in this protocol will also not result in additional costs to your health insurance or social security system.

The results of this study may not provide direct benefits to you, but they could benefit future patients.

Under Law 25.326, you have the right to access your data and request corrections if necessary. You may decide to withdraw from the study at any time by simply informing the research team through the contact numbers listed below or in person. If you decide to withdraw from the study, your data will not be used further, except for the information collected up until your withdrawal.

Treatment for Possible Damages or Complications

No harm or complications are expected from participating in this study, as the intervention will be minimal and non-invasive.

Contact and Questions

This protocol has been evaluated and approved by the Teaching and Research Committee and the Directorate of the ICR, authorizing its implementation.

For any questions regarding the study, you may contact:

Project Director: Lic. Leonardo Intelangelo

Researchers: Lic. Guillermo Andrés Mazzucco

Contact: Kinesiology Service at ICR or the Cardiorespiratory Research Unit (UDIKAR) of the Universidad del Gran Rosario (UGR).

Declaration of Consent

I have been informed about the purpose of this study, the procedures involved, and the potential risks and benefits.

I have had the opportunity to ask all my questions, and they have been answered satisfactorily.

I have been informed of whom to contact if I have further questions.

I have read this informed consent document and understand that I may withdraw from the study at any time.

Full Name of Volunteer: ________________________

Document Number and Type: ________________________

Signature: ________________________

Date: //___

Full Name of Researcher: ________________________

Document Number and Type: ________________________

Signature: ________________________

Date: //___

ANEXO 2

VARIABLES DE CATEGORIZACIÓN

Nombre:	Edad:	Genero: M / F	Talla: cm	Peso: Kg

IMC:	FEY:	OCD: SI / NO	NYHA: II – III - IV

TEST INCREMENTAL

PS vs CPAP vc GSSV

Fecha:

PRE TEST

SpO2:	Disnea:
FC:	Fatiga MI:
FR:	MV: CAF:
TA:	Confort:

TEST

TIEMPO	VEL	SpO2	FC	Disnea	Fatiga MI
1	3				
1	3,5				
1	4				
1	4,5				
1	5				
1	5,5				
1	6				
1	6,5				
1	7				
1	7,5				
1	8				

POST TEST

VAM:	TA:
SpO2:	Disnea:
FC:	Fatiga MI:
FR:	Confort:

RECUPERACIÓN (3´ post test)

SpO2:	TA:
FC:	Disnea:
FR:	Fatiga MI:

Fecha:

PRE TEST

SpO2:	Disnea:
FC:	Fatiga MI:
FR:	MV: CAF:
TA:	Confort:

TEST

TIEMPO	VEL	SpO2	FC	Disnea	Fatiga MI
1	3				
1	3,5				
1	4				
1	4,5				
1	5				
1	5,5				
1	6				
1	6,5				
1	7				
1	7,5				
1	8				

POST TEST

VAM:	TA:
SpO2:	Disnea:
FC:	Fatiga MI:
FR:	Confort:

RECUPERACIÓN (3´ post test)

SpO2:	TA:
FC:	Disnea:
FR:	Fatiga MI:

ANEXO 3:

TEST DE CARGA CONSTANTE

PS vs CPAP vc GSSV

Paciente (AyN):
ID:
Fecha:

PRE TEST

SpO2:	Disnea:
FC:	Fatiga MI:
FR:	MV: CAF:
TA:	Confort:

TEST

VAM:	Intensidad: km/h

TIEMPO	SpO2	FC	Disnea	Fatiga MI
1				
1				
1				
1				
1				
1				
1				
1				
1				
1				
1				
1				
1				
1				
1				
1				
1				
1				
1				
1				
1				
1				
1				
1				
1				
1				
1				
1				
1				
1				

POST TEST

T máx:	TA:
SpO2:	Disnea:
FC:	Fatiga MI:
FR:	Confort:

RECUPERACIÓN (3´ post test)

SpO2:	TA:
FC:	Disnea:
FR:	Fatiga MI:

Fecha:

PRE TEST

SpO2:	Disnea:
FC:	Fatiga MI:
FR:	MV: CAF:
TA:	Confort:

TEST

VAM:	Intensidad: km/h

TIEMPO	SpO2	FC	Disnea	Fatiga MI
1				
1				
1				
1				
1				
1				
1				
1				
1				
1				
1				
1				
1				
1				
1				
1				
1				
1				
1				
1				
1				
1				
1				
1				
1				
1				
1				
1				
1				
1				

POST TEST

T máx:	TA:
SpO2:	Disnea:
FC:	Fatiga MI:
FR:	Confort:

RECUPERACIÓN (3´ post test)

SpO2:	TA:
FC:	Disnea:
FR:	Fatiga MI:

ANEXO 4

ESCALA DE BORG MODIFICADA

0	NADA
0,5	MUY MUY LIGERA
1	MUY LIGERA
2	LIGERA
3	MODERADA
4	ALGO SEVERA
5	SEVERA
6	
7	MUY SEVERA
8	
9	MUY MUY SEVERA
10	MÁXIMA

ANEXO 5

VALORACIÓN DE GLOBAL DE CONFORT PRE TEST

A continuación quisiéramos saber cuán **confortable** se siente en éste momento con el sistema (con la CAF / MV).
Por favor señale en el cuadro el valor que más a se ajusta a su sentimiento:

0 MÁXIMO DISCONFORT - 10 MÁXIMO CONFORT

0	1	2	3	4	5	6	7	8	9	10

VALORACIÓN DE GLOBAL DE CONFORT POST TEST

A continuación quisiéramos saber cuán **confortable** se sintió durante la prueba con el sistema (con la CAF / MV).
Por favor señale en el cuadro el valor que más a se ajusta a su sentimiento:

0 MÁXIMO DISCONFORT - 10 MÁXIMO CONFORT

0	1	2	3	4	5	6	7	8	9	10
